# Predisposition and Environment: How Can They Be Governed?

**Published:** 1901-03

**Authors:** G. Alden Mills

**Affiliations:** New York


### PREDISPOSITION AND ENVIRONMENT: HOW CAN THEY BE GOVERNED?

BY DR. G. ALDEN MILLS, NEW YORK.

All the secretions which come in contact with the outward atmosphere must be subjected to sanitary conditions, otherwise they will produce disordered surroundings; left entirely without care, diseases will result. The teeth and mouth prove marked examples in proportion to the abnormal state of the body, through which these secretions are generated and have their flow. Every mouth that is left without sanitary care under these conditions will at once begin to accumulate what is termed "sordes," or what may be better understood by a portion of the public, "filth;" as the English say, "nasty." Polite as we may be, we, as practitioners, see cases that the latter term fully expresses. One oft-repeated phrase has been voiced in dental gatherings: "Could the teeth be kept absolutely clean, decay would find no field for its ravages." Too often this expression would seem to carry the impression that we are helpless. Therefore the result has been a laxity in definite emphasis for extreme efforts. We hardly dare to give our estimate of the percentage of practitioners who teach to their patients line upon line the necessity of assiduous, painstaking care of the teeth, yet we will say that it is marvellously small. To-day we are deluged with a multiplicity of devices in brushes, soaps, and washes; and last of all we have lately seen advertised by the railway in big letters, "Use Sapolio on the teeth." There is no doubt that it would clean teeth, and might not be a bad article to commence with in some cases. Is it not a fact that the dentist too often stops with these helpful remedies and too much the public is misled into the belief that these articles do prevent decay to such an extent that deception follows? That brushes and etceteras do to a degree lessen the possibilities of decay is true. Too much the patient is met, in answer to his inquiries regarding the saving of his teeth, by the wise answer, Nothing but a "prophylactic" treatment will save your teeth. And many a one draws a quicker and deeper breath and wonders, perchance, how much this is a bottle. We are not overdrawing a common way of looking at this question.

Now, while prophylaxis is quite a big term, yet it has a meaning if carried out on the line of intelligence, and this must be most largely by a practical demonstration on the patient. To get the needed results there must be an enlarged, systematic course of practice established, not by the few, but by the many. It is to the heart of our calling that this appeal must apply. I need not tell it here that there is a constantly increasing, retrograde nucleus fastening upon the legitimate calling of dentistry which has the commercial thought largely in view; therefore, there is a necessity for higher attainments on our side. We owe it to our clientele and ourselves to do all in our power to lessen the arduousness of labor. Prevention will prove a larger-meaning term than any other providing we do it with a will, endeavoring to prove its efficacy.

The remarks made by Dr. Jarvie, of Brooklyn, at a late meeting of the Odontological Society has inspired this paper to emphasize the subject, "Prevention of Caries." The doctor said a lady patient asked him why it would not be a good practical plan to have qualified persons come to our houses and clean teeth, as we have such persons come to cleanse and care for our nails? In connection with this there came another woman (dentist) the same day who had gone to the expense of legitimately preparing herself for the practice of dentistry. For ten months she had faithfully pursued the effort to gain a supporting clientele, until she was almost in despair at not having succeeded. This incident led him to propound the query whether it might not be a good idea to encourage the plan suggested by the lady. It was not surprising that it met with a chill, for, first, there would be a decided prejudice, and, as has been truly said, cleansing teeth is a big subject in its deepest application, as we have emphasized in the early part of this essay. This was suggested to Dr. Jarvie. I do think that good might come of the suggestion if but applied to the juvenile portion of the household, making it a part of the duty of nurses in the care of children, and I further think that the families of wealth and culture would welcome this kind of practice if endorsed by practitioners of good standing. Here is a suitably qualified person to inaugurate such a movement. For children to become habituated to the daily faithful care of the teeth would be a decided assistance to the dentist in the maturer care of the teeth, and in the line advocated there will be a growing appreciation of services rendered. Children who come into our hands, particularly the class we have been speaking of, treated by this preliminary training would become familiarized with a frequent manipulation, and this would render us valuable assistance in the more extended services required.

That this subject of systematic practice in the earlier care of teeth is to become a decidedly emphasized one I am sure is being outlined by a few sincere and earnest practitioners who are combining both the practical and the commercial, and by their proposition to their patients it presents a feature of novelty. It is evident that this will work favorably in more ways than are at first thought of. In the first place it will prove that dentistry may be made vastly more salvatory than ever before. It will lessen the labor of practice by making it quite unlikely and impossible for extreme operations to become necessary. It will do much to remove the dread of practice. Patients will become less resisting. There will be a large decrease in dental decay. The object of this course of practice is of a firm belief that teeth predisposed to caries will be the ones that we desire to improve. It stands to reason that the environment will be decidedly bettered. As it was once said by Dr. Foster Flagg, regarding his theory of the "new departure," "The teeth that most need saving will be the ones that will be saved." Familiarizing children with operative movements upon the mouth will attain results which can hardly be computed. These children will grow up into mature patients.

I have often asserted that a practitioner who would start out on these lines would never know a need for practice, and he will have a hold upon patients that will be difficult to break.

I have a student that spent four years in my office, during which period the effort was made to instil the convictions that the teachings on this line were sound; and he began to practise accordingly. It was a pleasure to notice the genial fellowship he had with his little patients. While his practice was conducted on lines of marked simplicity, yet the results were immensely practical. It is not claimed that our practice is wholly conducted on the systematic plan referred to, but the teachings and writings bear accord to the fact that there has been manifested a decided interest upon the subject throughout the entire practice, now nearing fifty years. In 1867 (I think), in the *Dental Register,* will be found an article on "Cleaning Teeth," that gave decided teaching regarding cleansing, and more particularly the value of polishing. Professor Cutler, at that date well known as a valued teacher, emphasized this article to the students of the Ohio Dental College as the best he had ever seen on the subject. There is a little history connected with the publication of this article. It was first sent to the *Dental Cosmos*, and after it had lain in the editorial sanctum for some weeks, hearing nothing from it, I wrote to know what to expect. The manuscript was returned with a note, thinking it hardly worthy of a place in the journal. I felt quite a little cut. The paper was shown to several dentists and their opinion asked as to its worthiness, and all said publish it. In this article the history was given of the first attention to the value of polishing teeth which was given by my preceptor. He said to me, "Do you know how nice a polish a natural tooth will take?" So he suggested that I should see what I could do with a six-year molar, just extracted. I took it to the laboratory and put it into a lathe, and with pumice and powder produced a brilliant surface. This tooth was carried in the pocket for a number of years, and often was exhibited to practitioners. In this paper these facts were stated, and the practice was emphasized and illustrated from a case on which several hours were spent. It was a patient that had had heroic treatment with iron remedies, and the teeth had been decidedly etched. I was solicited to do all I could to give smooth surfaces. At that time I exhibited the specimen tooth. The result was very satisfactory, and a fee of over one hundred dollars was paid, which at that time was thought to be large. I could have earned more filling teeth in the time taken.

We notice that Drs. Smith and Taylor term their services "massaging the teeth." Besides the polishing and cleansing, they manipulate the surfaces of the teeth, and, we think, the gums, which, no doubt, is productive of good results. No doubt Dr. Smith secures results that are decidedly salvatory, particularly on young teeth. It is a great oversight that so many of our practitioners have so long resisted a larger attention to this kindergarten practice. I am sure that this is our first duty to young children.

Our full duty will not be done on this line when we say we turn this practice over to assistants, unless they have been intelligently instructed. I am not quite sure but it would be the best way to familiarize the first-year students with the teeth and mouth. By this they would become used to the work, which is a very important beginning.

Dr. Taylor, of Hartford, has instituted a very commendable movement in the New York Dental School. He has sought to introduce the special instruction of this line of teaching by giving his own time to it and by offering a prize to the student who will show the best results. He began it at the last term and gave a prize. The prize is of little consequence, but the practitioner who becomes imbued with the value of this practice has a rich future before him of appreciation from those who get the demonstration of a definite practice of a systematic cleansing and polishing of teeth.

Dr. Smith's promises carry a large inducement to his patients,—viz., an immunity from decay that brings a necessity for filling to the minimum. Now, it is certain that he could not sustain such a plan if he could not prove it. We understand he is giving full proof.

The dealings of this paper have been wholly along the line of a juvenile practice, but be it far from my belief but that a very large percentage of salvatory benefits will accrue to the adult practice. While not a few come to us when much mischief has matured for the want of this juvenile practice, yet it stands to reason that a frequent attention would decrease the advance of disorder culminating in a larger mischief. As dentists we are not too much given to the exhibition of simple demonstrations. Simplicity leads us away from multiplicity. It has been said by some that they do not have difficult operations, for they first reduce the difficulties to easiness of operation. This is true of filling teeth.

The method advocated by Dr. Smith, as he gives it in detail as practised first on his little grandchildren, seems at first too simple, but it is a work commenced upon very impressible material which establishes a willing habit. We all know more or less the force of habit, particularly of unprofitable ones. This method of dealing with children will rob dental practice of its greatest bugbear, dread and fear. It is sure to establish an environment of bettered conditions that will stand over against the disabilities of predisposition. So far prophylactic treatment has alone been employed, by resorting to principally chemical agencies. If we can secure a larger favorableness of environment we will do much to lessen the need of chemical agencies. There is a condition of the body that is constantly exposed to sudden and unexpected changes, and we cannot eradicate them. There are many things that can be done which will lessen them. Our mental environment, our bodily environment, be they favorable or the opposite, will do much to help or hinder a favorable chemical equilibrium. These discords are the factors that operate so destructively by forming a union with atmospheric contact. We are brought face to face with the fact that there is nothing that can destroy the discord in physical conditions which give predisposition its lurking power of destruction. Now, most of all that we can do is to better the environment. While we seek to do this by working along prophylactic lines, it may be that there are some possibilities in constitutional treatment. There are those who are claiming that they do get better results. Dr. George Winkler is an ardent advocate of this practice. His writings are on record. Be this a remedial practice in any degree, it does not cover the ground nor interfere at all with the imperative needs of local treatment on the lines already referred to.

In connection with long familiarity with the treatment of the so-called "Riggs's Disease," I have often laid a good deal of stress upon the fact that the Riggs treatment meant also literally "cleaning teeth" from A to Z and from cutting edge to apex of root, if it so indicated a need. In many a severe case where this has been practised the results have revealed marvels of improvement, and in many cases, could the practice have been followed up, greater results would have accrued. In many cases that have come from places quite remote I have not had the opportunity to secure the best effects. The person has always been impressed with the importance of closely watching these cases. We have been told by practitioners who afterwards had the privilege of following up some of them, they did not have any encouragement because there was "no money in it;" "these people would go to New York and pay a big fee, but for them there were no funds left." Is not this a puerile excuse? Do these remarks justify the action? I think not. How were we able to get these "big fees"? When the results brought conviction I commenced to follow these by taking tuition, and putting into practice what I had learned. Is it to be supposed that one hundred dollars was received, more or less, for my *first* cases? No; all kinds of patients would let me practise on them to secure proficiency. This was twenty years ago.

In other lines of practice operators are supposed to get somewhat skilful in this length of time if ever. During this period I have had a variety of experiences that come more or less to those that enter new fields of practice. Scarcely any one was emphasizing Dr. Riggs's method of practice, as dealing systematically with the disorder so much talked of now.

Providentially, I believe, I was brought in personal contact with the late doctor, and during some thirteen months a continued observation of cases before and after deepened a decided conviction on my mind regarding the value of what was seen. In this connection all I saw and heard emphasized this initial practice of "cleaning teeth among the juvenile patients." Dr. Taylor at one time associated with Dr. Riggs and imbibed the virtue of such practice, and for the larger carrying out of what he learned and has in some small degree practised he sees a larger field for its usefulness; and becoming interested by observation at Dr. Smith's chair, he combines methods and goes out to teach them among those that are to make up to some extent the future profession. We trust many other dental institutions will hasten to follow the good example.

We have heard some little carping about the commercial spirit embraced in Dr. Smith's contract system. Such an objection is a puerile one. I find that patients that make contracts, and make even part payment, become more interested as participants in what is being done for them. This is a well-known fact and often commented upon concerning plate-work. It works admirably in the plan of putting the mouth in order and surgical treatment. It has a marked effect on both patient and operator to get the money question behind and out of the way, and there would be less loss of funds for faithful services rendered. It is a severe disappointment to lose compensation for hard-earned rewards, certainly when it is meanly imposed upon us. The late Dr. Atkinson had a bitter and depressing experience on this line. Over ninety-odd thousands of hard-earned dollars was a large amount to leave behind at one's demise. Predisposition to meanness inborn in many natures requires also a much-bettered environment.

This subject alone is worthy of attention as an advance in professional dealing.

